# Barriers to sexual and reproductive health services among Albanian university students

**DOI:** 10.3389/frph.2025.1637583

**Published:** 2026-02-09

**Authors:** Jonila Gabrani, Lumturi Merkuri, Voltisa Gjergji, Kristi Cela, Iva Rrugia, Rovena Lika Kushta

**Affiliations:** Center for Scientific Research in Public Health, Faculty of Medical Technical Sciences, Universiteti Europian I Tiranes, Tirana, Albania

**Keywords:** Andersen's behavioral model, barriers & facilitative factors, sexual and reproductive health (SRH), university students, Western Balkan countries

## Abstract

**Background:**

Access to sexual and reproductive health (SRH) services remains limited among university students in Albania, despite global progress in HIV/STI prevention. Low awareness, stigma, and fragmented youth-friendly services continue to hinder preventive healthcare utilization. Understanding the factors shaping SRH-seeking behavior is essential for designing effective interventions.

**Methods:**

A cross-sectional study was conducted among 7,679 students from public and private universities in Albania (2024–2025). A structured, validated questionnaire assessed SRH knowledge, awareness of testing locations, condom access, and use of preventive services. Descriptive statistics, chi-square tests, and multivariable logistic regression were performed to identify predictors of HIV/STI testing, guided by Andersen's Behavioral Model.

**Results:**

SRH service utilization was low, with only 11.4% of students reporting STI screening and 7.6% HIV testing. Students who were aware of testing locations had significantly higher odds of ever being tested (OR = 7.52; 95% CI: 6.21–9.09). Gender differences were pronounced, female students were more likely to report condom non-use and uncertainty about access points. Although sexual health education was associated with testing in bivariate analyses, only parental communication remained significant in the adjusted model.

**Conclusions:**

Significant gaps in SRH awareness, access, and preventive healthcare engagement exist among Albanian university students. Enabling factors, particularly knowledge of service availability, play a greater role in influencing utilization than individual or predisposing characteristics, consistent with Andersen's Behavioral Model. Strengthening youth-friendly SRH services, increasing the visibility of testing sites, integrating SRH education into university programs, and addressing gender-specific barriers are essential to improve uptake of preventive SRH services in Albania.

## Introduction

Despite global efforts to improve STI and HIV prevention, young adults continue to exhibit low engagement with preventive healthcare, including STI screening and routine gynecological visits (1). Sexual and reproductive health (SRH) is a fundamental aspect of overall well-being, yet access to essential SRH services remains a significant challenge for university students in Albania. Barriers such as misinformation, stigma, and limited awareness of service availability contribute to the underutilization of healthcare resources among this demographic (2).

Understanding these barriers is essential for designing effective interventions to improve SRH outcomes among university students. This study directly addresses these gaps through a detailed examination of students' knowledge, awareness, and use of essential SRH services in Albania. It investigates individual drivers of behaviour (such as SRH education, condom use, and communication patterns) as well as system-level barriers, including awareness of testing locations and the accessibility of services. Through this analysis, the study identifies the key determinants that shape preventive healthcare utilisation in this population.

### Access to sexual and reproductive health services in Albania

In Albania, a Southeast European country with a transitioning health system, SRH services are theoretically accessible through public health institutions, with basic preventive services, including STI and HIV testing, offered at low or no cost. Studies in Albania highlight that public facilities often lack youth-friendly environments, discouraging students from seeking STI testing or contraception (2). Studies consistently show low HIV/STI testing rates among young adults. Limited awareness of available services, coupled with the lack of adequate infrastructure and bureaucratic hurdles, make it difficult for youth to navigate and access SRH services in Albania (3). The lack of integrated SRH services in universities further restricts access, particularly for students with limited financial means. However, the reality is that many students do not engage with these services. Cultural stigma surrounding sexual health, insufficient integration of SRH education in university curricula, and low levels of awareness about available services contribute to this underutilization. Further complicating access, the private healthcare sector in Albania has expanded significantly in urban centers, offering a broad range of SRH services. While these services may be perceived as higher quality or more confidential, they often require out-of-pocket payments, limiting access for students with financial constraints (4).

Despite policy improvements, university students in Albania and the Western Balkan region face significant obstacles in accessing sexual and reproductive health services. Many students report that public healthcare facilities are not welcoming to young adults seeking sexual health services (5). There is also fear of judgment from medical professionals discourages students from seeking STI testing and contraception. During the COVID-19 pandemic, access to contraception and reproductive health counseling was further disrupted across the Western Balkans, leading to an increase in unprotected sex and reduced STI screenings. Economic barriers prevent consistent use of birth control and STI checkups, particularly among students in public universities (6–8).

### Availability of SRH services

The availability of sexual and reproductive health services in the region varies significantly by geographical location, economic status, and university affiliation (public vs. private). Public and universities in Albania and the Western Balkans often lack dedicated SRH centres or youth-friendly clinics, leaving students with limited access to contraceptives, STI screening, and reproductive health counselling (9). In rural and peripheral areas, the availability of SRH services is extremely limited, forcing students to travel long distances to access reproductive healthcare. A survey in Albania found that a third of participants expressed discomfort inquiring about SRH matters, while nearly two-thirds rarely or never sought SRH services from a doctor or clinic (3). Studies suggest that individual factors such as gender, age, and prior exposure to SRH education influence health-seeking behaviour, including the likelihood of utilising preventive services such as STI screening and contraceptive counselling (2). However, there remains a gap in understanding how these factors interact with perceived barriers and health system characteristics to shape SRH service utilisation among Albanian university students.

This study aims to explore the barriers that hinder SRH service access among university students in Albania and to identify key sociodemographic and educational factors influencing preventive healthcare utilization. Understanding these patterns is crucial for developing targeted, youth-friendly interventions and policy reforms to improve SRH outcomes. This targeted approach links the broader SRH challenges described in the literature with specific, measurable behaviours and access barriers experienced by Albanian university students.

## Methods

### Conceptual framework on access to SRH services

Good access to SRH services is a key determinant for improving utilization among young adults. Access is a complex and multidimensional concept that has been modelled in several theoretical frameworks. One of the most influential is Andersen's Behavioural Model, which categorises determinants of health service utilisation into predisposing factors (e.g., age, gender, social structure), enabling factors (e.g., healthcare availability, affordability), and need factors (e.g., perceived health status, symptoms) (10). Recent elaborations of access further highlight five abilities that populations require to interact effectively with healthcare services: the ability to perceive the need for care, to seek care, to reach services, to pay for them, and to engage in care processes.

**Figure 1:**
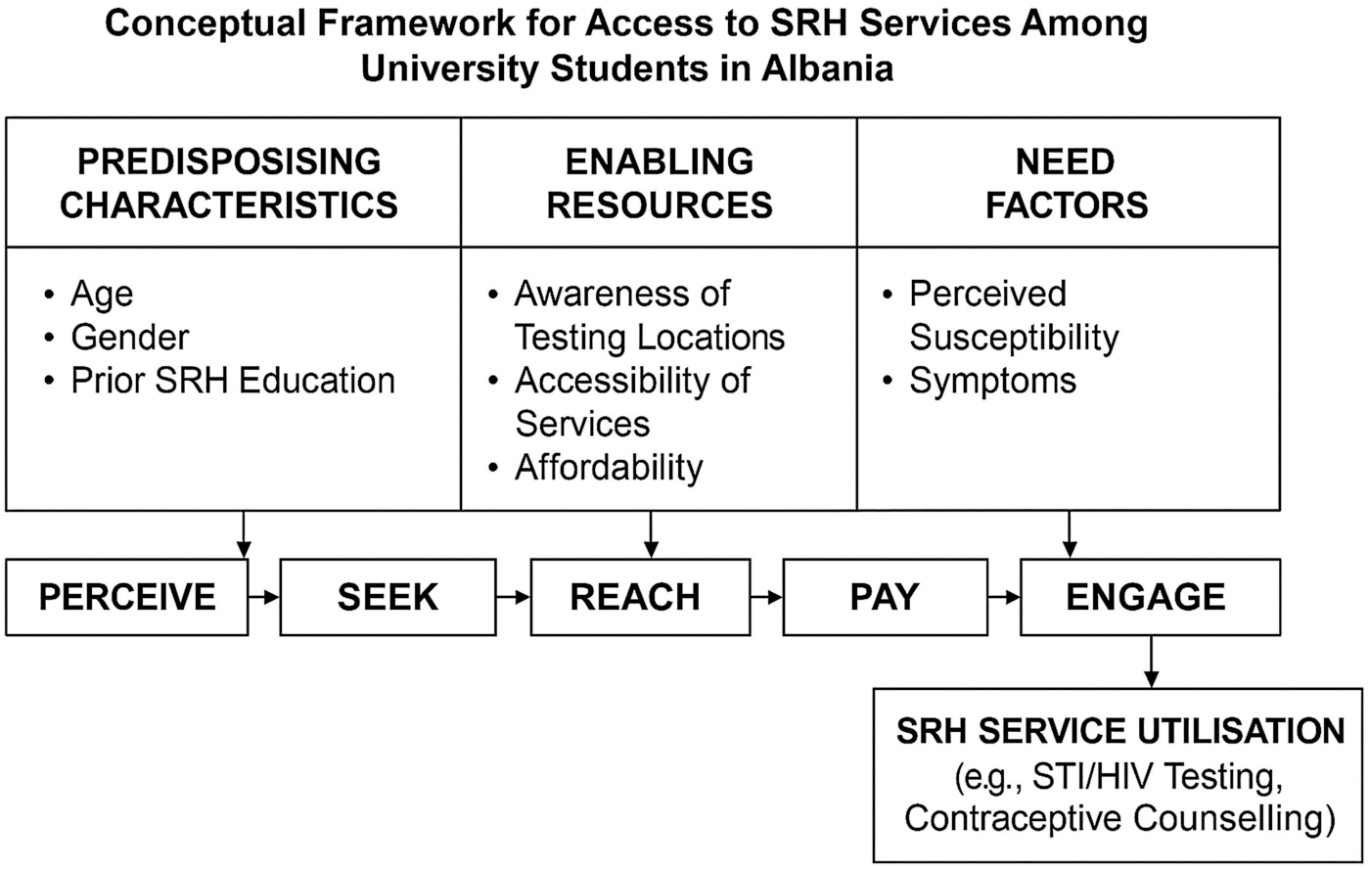
Conceptual framework for access to SRH services among university students in Albania. This framework combines Andersen's Behavioral Model of Health Services Use with the five abilities of access (perceive, seek, reach, pay, engage). It illustrates how predisposing characteristics, enabling resources, and need factors influence awareness, accessibility, and ultimately utilisation of SRH services, including STI/HIV testing and contraceptive counselling. Based on Andersen, R. M. (1995). Revisiting the Behavioral Model and Access to Medical Care: Does it Matter? Journal of Health and Social Behavior, 36(1), 1–10. doi: 10.2307/2137284.

To enhance readability and provide a clearer overview of the determinants of SRH service utilisation examined in this study, we developed a graphical representation of the conceptual framework (Figure 1). The figure integrates Andersen's Behavioral Model with the five access abilities (perceive, seek, reach, pay, engage), adapted from contemporary access literature. This framework illustrates how predisposing factors (e.g., age, gender, prior SRH education), enabling factors (e.g., awareness of testing locations, affordability), and need factors (e.g., perceived susceptibility or symptoms) interact to shape students' utilisation of SRH services.

### Study design and data collection

This descriptive cross-sectional study was conducted between September 2024 and February 2025 to explore the barriers to accessing sexual and reproductive health (SRH) services among university students in Albania. Data were collected from a representative sample of students enrolled in public and private universities across the country during the 2024–2025 academic year.

A mixed-method approach was employed, combining quantitative and qualitative data collection techniques. The questionnaire was developed based on WHO SRH and HIV/STI surveillance instruments and adapted from previously validated tools used in similar university-based studies. Content validity was assessed through expert review by three public health researchers and two SRH practitioners in Albania. The survey was pretested with 50 students to assess clarity, comprehension, and appropriateness of items. Minor revisions were made to improve wording and response options. The questionnaire consisted of three main sections: (1) socio-demographic data, (2) knowledge and attitudes regarding SRH, and (3) sexual practices and use of SRH services.

Qualitative insights were gathered through Focus Discussion Groups (FGDs) with 10–12 participants each, held in five selected universities. These discussions aimed to explore students' perspectives on SRH in an interactive setting. Thematic analysis was conducted to identify recurring themes and nuanced opinions. The qualitative analysis is part of another upcoming publication.

### Study population

The target population included students from all accredited Higher Education Institutions (HEIs) in Albania, based on the list published by the Agency for Quality Assurance in Higher Education (ASCAL). Students who provided informed consent were included in the study; those who declined to participate were excluded. A stratified sampling approach was used to ensure representation across public and private universities and across all major academic disciplines. Within each institution, students were recruited through faculty coordinators who distributed the online questionnaire to all enrolled students. Although the survey was open to all students, the stratification ensured proportional representation by university type and academic year. A total of 7,679 students completed the questionnaire, yielding an estimated response rate of approximately 62% across institutions.

### Data collection procedure

Data was collected electronically via Google Forms. Permissions were secured from university rectorates, and focal points at each university supported students during survey completion to ensure accuracy and clarity.

### Data analysis

Data was analyzed using SPSS version 29. Descriptive statistics (frequencies, percentages, means, standard deviations) and inferential analyses (Student's *t*-test, Chi-square test, Odds Ratios with 95% Confidence Intervals, and *p*-values <0.05) were applied to assess associations and determine statistical significance. For bivariate analyses, Pearson's chi-square tests were used to examine associations between categorical predictors and HIV/STI testing outcomes. Effect sizes were reported using Cramer's V. Logistic regression was conducted to identify independent predictors of STI testing. Model assumptions, including multicollinearity (VIF <2.0), linearity in the logit for continuous predictors, and absence of influential outliers, were checked prior to final modelling. Model fit was evaluated using the Omnibus Test, −2LL, Nagelkerke R², and the Hosmer–Lemeshow test, and classification accuracy was reported. All tests were two-tailed with significance set at *p* < 0.05. Findings were presented in tables and graphs to illustrate trends in SRH knowledge and behaviors.

### Ethical considerations

The study adhered to the Declaration of Helsinki for ethical research involving human subjects. Ethical approval was granted by the Ethics Board of the European University of Tirana Nr. 6 protocol date 10.06.2024. Participants received detailed information about the study's purpose, assured confidentiality and anonymity, and retained the right to withdraw at any time.

## Results

### Demographic characteristics

The study participants were predominantly young, with 90.9% of them aged 18–25 (Table 1). The majority were single (61.5%) or in a causal relationship (20.8%).

**Table 1:** Demographic data of university students.

Demographic characteristics	Frequency	Percentage
Public University	4,694	61
Private University	2,985	39
Age		
18–20 years old	4,891	63.7
21–25 years old	2,090	27.2
Over 25 years old	698	9.1
Marital status		
Cohabitation	716	9.3
Single	4,720	61.5
Divorced	59	0.8
Married	590	7.7
In a casual relationship	1,594	20.8

The data includes students from a wide range of educational institutions, showing a good distribution of data between public and private universities. First-year students make up the largest group of participants (38.9%). This result is understandable given that first-year students are often more engaged in new activities, including participating in surveys.

### Access barriers and utilization of SRH services

Overall, the findings reveal significant gaps in access to sexual and reproductive health services, particularly in STI and HIV/AIDS testing (Tables 2, 3). Only 11.4% of students have been tested for STIs, and even lower 7.6% have undergone HIV/AIDS testing, indicating a severe lack of engagement with preventive health measures.

**Table 2:** STI and HIV/AIDS testing among university students A. Bivariate associations (chi-square tests).

Outcome	Predictor	Group	Tested (%)	Not tested (%)	Total *n* (%)	χ²	*p*-value
HIV testing	Gender	Male	11.5%	88.5%	1,713 (22.3%)	46.83	<.001
		Female	6.5%	93.5%	5,966 (77.7%)		
	Sexual education	No	6.9%	93.1%	5,734 (74.7%)	17.70	<.001
		Yes	9.8%	90.2%	1,945 (25.3%)		
STI testing	Gender	Male	11.5%	88.5%	1,713 (22.3%)	17.73	<.001
		Female	6.5%	93.5%	5,966 (77.7%)		
	Sexual education	No	10.5%	89.5%	5,734 (74.7%)	19.43	<.001
		Yes	14.1%	85.9%	1,945 (25.3%)		

**Table 3:** STI and HIV/AIDS testing among university students B. Multivariable logistic regression (adjusted odds ratios).

Outcome	Predictor	aOR	95% CI	*p*-value
HIV testing	Gender (Male)	0.552	0.460–0.662	<.001
	Sexual Education (Yes)	1.525	1.271–1.830	<.001
STI testing	Gender (Male)	0.740	0.630–0.868	<.001
	Sexual Education (Yes)	1.450	1.243–1.691	<.001

Tables 1–3 present the bivariate associations between gender, university type, sexual health education, and HIV/STI testing. Significant disparities emerge across all three predictors. Males consistently reported higher testing rates than females for both HIV (11.5% vs. 6.5%) and STIs (11.5% vs. 6.5%), indicating gendered differences in testing behaviours. Likewise, prior exposure to sexual health education was strongly associated with higher testing rates. These results highlight the importance of both individual characteristics and institutional environments as determinants of SRH service use.

A major contributing factor is the lack of awareness, with 53.8% of students (4,129 individuals) unsure about where to get tested (Tables 4, 5).

**Table 4:** Awareness of testing locations.

Awareness of testing centers	Percentage (%)
Do not know where to get tested	53.8
Know where to get tested	46.2

**Table 5:** Association between awareness of testing locations and HIV/STI testing behavior.

Outcome	Awareness group	Tested *n* (%)	Not tested *n* (%)	Total *n* (%)	χ²(df = 1)	*p*-value	Cramer's V
HIV testing	Know where to get tested	531 (15.0%)	3,019 (85.0%)	3,550 (46.2%)	502.77	<.001	0.256
	Do not know	55 (1.3%)	4,074 (98.7%)	4,129 (53.8%)	–	–	–
STI testing	Know where to get tested	736 (20.7%)	2,814 (79.3%)	3,550 (46.2%)	570.17	<.001	0.272
	Do not know	139 (3.4%)	3,990 (96.6%)	4,129 (53.8%)	–	–	–

HIV/AIDS testing rates are notably lower than STI testing, reflecting deep-seated stigma and misconceptions surrounding HIV screening. This could indicate a lack of education on the importance of routine HIV testing as well as fear of positive results and societal discrimination.

More than half (53.8%) of students do not know where to get tested for STIs or HIV/AIDS. This lack of awareness suggests that students may not seek testing due to limited knowledge of available services. There is an urgent need for health campaigns to educate students on where and how to access sexual health services.

Tables 4, 5 underscores the central role that awareness plays in students' testing behaviours. Awareness of testing locations showed one of the strongest associations in the study. Students who knew where to access HIV testing were more than eleven times as likely to have been tested compared to those without this knowledge (15.0% vs. 1.3%), and a similar pattern was seen for STI testing (20.7% vs. 3.4%). These large effect sizes (Cramer's V = 0.256–0.272) indicate that knowledge of service availability is not merely correlated with testing but represents a critical enabling factor influencing utilisation. Increasing visibility of testing locations on campuses may therefore be one of the most impactful strategies to improve testing uptake.

Condom accessibility also presents challenges (Table 6). While 60.5% of respondents purchase condoms from pharmacies, only a small fraction (0.5%) obtains them from family planning centers, despite the availability of free or low-cost services. This suggests a lack of awareness or discomfort in accessing reproductive health facilities. Additionally, 33.1% of students do not use condoms at all, increasing their risk of STIs and unintended pregnancies. Alarmingly, 2.9% do not know where to obtain condoms, highlighting another gap in sexual health knowledge and access.

**Table 6:** Condom access sources by gender.

Source of condoms	Female (%)	Male (%)	Total *n* (%)	χ² (df = 4)	*p*-value	Cramer's V
Pharmacy	69.6	30.4	4,642 (60.4%)			
Do not use condoms	94.0	6.0	2,549 (33.2%)			
Store/Supermarket	55.1	44.9	234 (3.0%)	635.8	<0.001	0.28
Do not know where to obtain them	82.6	17.4	219 (2.8%)			
Family planning center	82.9	17.1	35 (0.5%)			

Gender differences in condom access were substantial (Table 6). Females were disproportionately represented among students who reported not using condoms (94.0%), not knowing where to obtain them (82.6%), or relying on family planning centres (82.9%). In contrast, males more commonly obtained condoms from pharmacies or supermarkets. The significant association (χ² = 635.83, *p* < .001) suggests not only behavioural differences but also possible disparities in comfort, access autonomy, and gendered social norms influencing contraceptive practices. These findings point to a need for gender-sensitive interventions that address structural and cultural factors shaping condom access among female students.

The results indicate a strong correlation between knowledge of STI testing locations and actual testing behavior. Among those who know where to get tested, 20.7% have undergone STI testing. Among those who do not know where to get tested, only 3.4% have been tested. More than half (53.8%) of students is unaware of testing locations, representing a major barrier to STI screening (Table 7).

**Table 7:** Cross tabulation: knowledge of STI testing locations and testing behavior.

Knowledge of testing locations	Tested (%)	Not Tested (%)	Total *n* (%)	OR (95% CI)	*p*-value
Know where to get tested	736 (20.7%)	2,814 (79.3%)	3,550 (46.2%)	7.52 (6.21–9.09)	<0.001
Do not know	139 (3.4%)	3,990 (96.6%)	4,129 (53.8%)	Ref.	–

Statistical Significance: Pearson Chi-Square (χ² = 570.169, df = 1, *p* < 0.001), Fisher's Exact Test (*p* < 0.001). The *p*-value confirms a statistically significant relationship between knowing where to get tested and undergoing STI testing.

This highlights the urgent need for awareness campaigns about where and how students can access STI testing. The data suggests that lack of knowledge may be a stronger barrier than fear or stigma, preventing students from utilizing available services. Table 7 confirms that students who knew where STI testing services were located were dramatically more likely to be tested. Awareness increased the odds of testing by more than seven-fold, representing the strongest predictor in the unadjusted analyses. This finding reinforces the importance of service visibility and suggests that universities and public health institutions need to prioritise clear, stigma-free communication about where and how students can access SRH services.

### Predictors of STI testing

The regression model confirms that students who received sexual health education, have open discussions with parents, or regularly use condoms are significantly more likely to undergo STI testing (Table 8). These results emphasize the urgent need for targeted interventions to improve awareness, access, and utilization of sexual health services. Educational initiatives should focus on informing students about STI/HIV testing locations, reducing stigma, and encouraging condom use as a primary preventive measure. Addressing these barriers is essential for enhancing students' sexual and reproductive health outcomes and ensuring they make informed, responsible decisions. A significant 68.5% of students have never discussed sexual health with a doctor, while only 31.5% have engaged in such discussions.

**Table 8:** Regression analysis: predictors of STI testing.

Predictor	B	SE	OR	95% CI for OR	*p*-value
Sexual health education in school	−0.001	0.033	0.999	0.94–1.07	0.967
Condom acquisition source	−0.033	0.064	0.97	0.86–1.10	0.608
Discussed sexual health with parents	−0.794	0.081	0.45	0.39–0.53	<0.001
Condom use at last intercourse	0.116	0.084	1.12	0.95–1.32	0.167
Constant	−0.139	1.749	0.87	–	0.937

Key predictors of STI Testing (*p* < 0.001): Sexual health education in school, Source of condom acquisition, Discussion of sexual health with parents, Use of a condom in the last sexual encounter.

The full logistic regression model predicting STI testing was statistically significant and demonstrated acceptable classification accuracy (81.1%). However, the model explained a modest proportion of variance (Nagelkerke R² = 0.037), suggesting that although the included predictors contribute to STI testing behaviour, additional unmeasured factors likely play important roles. Among the four predictors, only parental communication significantly influenced testing behaviour: students who had not discussed sexual health with their parents had more than twice the odds of having undergone STI testing. This counterintuitive result aligns with evidence from regional studies indicating that university students may seek testing confidentially when they lack open communication at home. Sexual health education, source of condom acquisition, and condom use during last sexual activity did not independently predict testing after statistical adjustment.

## Discussion

### Healthcare access and utilization of SRH services, STI and HIV testing

Access to sexual and reproductive health (SRH) services among Albanian youth, particularly university students, faces several challenges, including limited knowledge, accessibility issues, and underutilization of available services.

The findings of this study indicate critical gaps in students' knowledge, access, and utilization of sexual and reproductive health (SRH) services, particularly in STI/HIV testing and contraception. The low rates of STI testing (11.4%) and HIV/AIDS testing (7.6%) reflect a lack of engagement with preventive healthcare, likely due to limited awareness, stigma, and fear of discrimination. More than half of students (53.8%) do not know where to get tested, which suggests that a lack of information is a more significant barrier than stigma alone. Utilization rates of SRH services among university students are notably low. Research indicates that 78% of sexually active students did not use Voluntary Counseling and Testing (VCT) services. Factors contributing to this underutilization include stigma, lack of awareness, and perceived social norms. Students from rural areas and those in their first academic year are less likely to use HIV services, suggesting disparities based on geographic and educational backgrounds.

When compared to regional studies, these findings align with patterns observed in the Western Balkans, where low HIV testing rates have been attributed to stigma, misinformation, and limited access to youth-friendly health services. A study in Kosovo found that only 15% of young people had been tested for STIs, with stigma and lack of awareness cited as key barriers (11). Another study conducted among university students in Northern Kosovo revealed that only 5.4% had been tested for HIV, despite 70.9% expressing a positive attitude towards testing. Factors such as limited awareness, stigma, and insufficient access to youth-friendly health services were identified as significant barriers (12). Similarly, research from North Macedonia revealed that fewer than 20% of university students had sought STI testing, despite increased sexual activity (13). These comparisons suggest that Albania faces similar challenges to its regional counterparts and requires targeted interventions to improve access and knowledge about SRH services.

A statistically significant correlation (*p* < 0.001) was found between knowing where to get tested and undergoing STI testing, emphasizing that awareness is a strong predictor of testing behavior. Among those who know where to get tested, 20.7% had undergone STI testing, while among those unaware, only 3.4% had been tested. This suggests that increasing awareness through health education campaigns could significantly improve testing rates. Studies in Serbia and Bosnia and Herzegovina have also reported low levels of STI screening due to limited youth-friendly services and social stigma (14, 19).

Condom use patterns further reflect accessibility challenges and gender differences in contraceptive responsibility. While 60.5% of students purchase condoms from pharmacies, only 0.5% obtain them from family planning centers, despite these centers offering free or low-cost services. This suggests that students either lack awareness of available resources or feel uncomfortable accessing them. The finding that 33.1% of students do not use condoms at all highlights high-risk behaviors, reinforcing the need for enhanced sexual health education and awareness campaigns. Research in Montenegro and Albania (15, 16) confirms similar trends, where low condom use is influenced by gender norms, misconceptions, and limited accessibility to SRH services.

Despite the existence of family planning centers and counseling services integrated into Albania's public health system, accessibility remains a concern. Factors such as inadequate infrastructure, bureaucratic hurdles, and financial constraints hinder effective service delivery. The Institute of Public Health's capacity to coordinate the Contraception Logistics Management Information System (CLMIS) is perceived as insufficient due to staffing and financial issues, leading to inadequate monitoring and supervision of SRH services (17).

Gender differences in SRH behaviour were pronounced and are consistent with broader patterns observed in Albania and the Western Balkans. Female students were significantly more likely to report not using condoms, uncertainty about where to obtain them, and lower uptake of HIV/STI testing. These findings may reflect entrenched gender norms in the Albanian context, where women often bear greater social scrutiny regarding sexual activity and may experience stronger stigma when seeking SRH services. Qualitative studies in the region have documented that young women frequently avoid purchasing condoms due to concerns about judgment, while young men more often assume responsibility for contraceptive procurement. These cultural expectations may limit women's autonomy in contraception use and reduce their comfort accessing preventive services. Addressing these gender-specific barriers is essential for designing equitable, youth-friendly SRH interventions.

Although this manuscript focuses on quantitative findings, complementary qualitative data collected through five focus group discussions are being analysed in a companion paper. Preliminary themes highlight stigma, uncertainty about confidentiality, and discomfort seeking information from providers, all factors that reinforce the quantitative patterns we report. These qualitative insights will further contextualize the barriers identified here and deepen the understanding of students lived experiences with SRH services.

## Conclusions

This study underscores the critical need to address barriers to accessing sexual and reproductive health (SRH) services among Albanian university students. The findings highlight the pivotal role of awareness, education, and stigma reduction in enhancing healthcare-seeking behaviors.

These findings align closely with the Andersen Behavioral Model. *Enabling factors*, particularly knowledge of where to access services, played a far greater role in predicting utilization than individual *predisposing factors* such as gender or sexual health education. The adjusted model also showed that *need-related* factors (such as communication with parents about sexual health) continued to influence behaviour, underscoring how interpersonal dynamics shape perceived need for care.

The results suggest that improving enabling conditions (such as visibility of testing sites, youth-friendly service design, and gender-sensitive access points) may have the greatest impact on increasing preventive SRH engagement. Strengthening these access pathways directly supports the Andersen model's core assertion that utilisation improves when individuals not only perceive the need for care but also know how, where, and feel able to obtain it.

A substantial barrier identified was the lack of awareness regarding the availability of STI and HIV testing services, with more than half of the surveyed students indicating uncertainty about where such services could be accessed. This gap in awareness significantly hindered students' engagement in preventive healthcare practices. Furthermore, the study revealed that students who had received formal education on SRH topics were notably more proactive in seeking preventive care, including STI screening and routine gynecological check-ups. Although sexual health education and university type were associated with testing in bivariate analyses, only parental communication remained a significant predictor in the adjusted model, indicating that structural and informational barriers exert a stronger influence than individual behaviours alone. This aligns with previous research, reinforcing the importance of education and open communication in family (18).

Condom access also revealed gendered disparities, with female students disproportionately reporting non-use; patterns that point to clear inequities in comfort, access, and autonomy related to contraceptive practices.

Despite a high prevalence of sexual activity among respondents, overall participation in preventive healthcare services remained disappointingly low, representing a missed opportunity for timely diagnosis, intervention, and health promotion.

To mitigate these challenges, it is essential to promote awareness of STI/HIV testing services through university health centers, digital outreach, and peer-led initiatives. Incorporating clear, accessible information about testing locations and procedures into sexual health education curricula can further bridge this gap. Public health messaging should aim to normalize STI screening by framing it as a routine and essential component of general health maintenance. Simultaneously, improving the accessibility of SRH services by enhancing infrastructure, streamlining administrative procedures, and ensuring sufficient funding is crucial for equitable service delivery.

## Data Availability

The raw data supporting the conclusions of this article will be made available by the authors, without undue reservation.
